# Tackling Loneliness, Ineffective Social Support, and Mental Ill‐Health Among People With Higher Weight

**DOI:** 10.1111/hex.70192

**Published:** 2025-03-27

**Authors:** Joanne A. Rathbone, Tegan Cruwys, Kate A. B. Western, Jessica L. Donaldson, Catherine Haslam, Elizabeth Rieger, Fiona Tito Wheatland, Paul Dugdale

**Affiliations:** ^1^ School of Medicine and Psychology Australian National University Canberra Australian Capital Territory Australia; ^2^ School of Psychology University of Queensland St Lucia Queensland Australia; ^3^ Health Care Consumers Association (ACT), Chifley Health and Wellbeing Hub Chifley Australian Capital Territory Australia

**Keywords:** group psychotherapy, mental health, social isolation, weight stigma, well‐being

## Abstract

**Background and Objectives:**

People with higher weight are at greater risk of experiencing loneliness and mental ill‐health, in part due to challenging social networks that can be unsupportive of efforts to engage in positive health behaviours and a source of weight‐based stigma and discrimination. Targeting this issue is a manualised intervention, Groups 4 Health (G4H), that helps people to optimise social connectedness and group‐based belonging for effective support to reduce loneliness and mental ill‐health. We evaluated the efficacy of this program for people with higher weight.

**Design and Participants:**

We conducted a matched controlled trial (non‐randomised) with 98 Australian adults with a body mass index (BMI) ≥ 30 kg/m^2^ (*n* = 49 in the intervention group). Retention rates at T2 were comparable to previous trials: intervention group *n* = 33 (67.3%); matched control group *n* = 36 (73.5%).

**Intervention and Outcomes:**

Loneliness, well‐being, weight‐related social support, depression, and eating disorder symptoms were assessed pre‐intervention (T0), post‐intervention (T1), and at 4‐month follow‐up (T2).

**Results:**

Among the intervention group, loneliness (*d* = −0.66, *p* < 0.001), depression (*d* = −0.58, *p* < 0.001), and eating disorder symptoms (*d* = −0.77, *p* < 0.001) all significantly decreased from T0 to T2. Similarly, well‐being (*d* = 0.80, *p* < 0.001) and experiences of effective weight‐related social support (*d* = 0.68, *p* < 0.001) significantly increased from T0 to T2. These positive changes were not observed in the matched control group.

**Conclusions:**

The findings provide strong preliminary support for the efficacy of G4H among people with higher weight to address loneliness and challenging social networks, which pose key psychosocial barriers to health.

**Patient and Public Contribution:**

The Groups 4 Health program has previously undergone a published codesign and consumer feedback process. The materials for this study were co‐produced with a member of the research team with lived experience to ensure that the content was non‐stigmatising and relevant to the population of the study. The research team member is a representative of a consumer advocacy association, and contributed to the study design, data collection, interpretation of results, and manuscript revisions.

## 1 Introduction

People with higher weight (body mass index [BMI] ≥ 30 kg/m^2^) experience many challenges that undermine their mental and physical health, engagement in positive health behaviours, and capacity to achieve and sustain weight loss. (The term ‘people with higher weight’ is used throughout this article and the conduct of this research, as terms like ‘people with obesity’ or ‘people with overweight’ are often experienced as stigmatising by those with a BMI ≥ 30 kg/m^2^. The preference for using ‘people with higher weight’ was shared by a member of the research team with lived experience of higher weight and weight stigma.) Among these, loneliness, characterised by feelings of disconnection from others and a lack of belonging [1], features prominently [2, 3, 4]. This is in part due to challenging social networks that are a source of stigma and discrimination, and are often ineffective in supporting positive health behaviour change [5, 6]. Addressing this issue is the Groups 4 Health (G4H) psychotherapy program, designed to reduce loneliness by building and sustaining effective group‐based support and belonging. The current study provided the first evaluation of G4H for people with higher weight.

Loneliness has been identified as a serious threat to both mental and physical health, increasing risks of depression, anxiety, suicidality, substance use, inflammatory processes, and all‐cause mortality [7, 8]. Previous research has also found that loneliness is associated with reduced engagement in physical activity in the general population [9]. While this relationship is likely bidirectional, the effect of loneliness on physical activity is stronger than the reverse, such that loneliness undermines future engagement in physical activity. Loneliness therefore presents a substantial barrier to health for people with higher weight. Indeed, meta‐analyses suggest the risk of loneliness to overall health is likely to be greater than the risk posed by higher weight directly [10].

Part of the reason why people with higher weight are at an increased risk of experiencing loneliness is because they face pervasive stigma and discrimination from both wider society and their own social networks [11, 12]. Experiencing weight‐based stigma and discrimination has been associated with decreased motivation and self‐efficacy to engage in healthy eating and physical activity [13, 14]. Stigma also increases the risk of developing mental ill‐health, including depression and disordered eating [5]. People with higher weight most often experience discrimination from those closest to them: family, romantic partners, and friends account for up to 82% of these experiences [12]. These experiences undermine a sense of connection and belonging [2, 15], which limits access to social support. Social support is an important resource for promoting both general health and weight management [16]. Effective social support—particularly from family members, romantic partners, and friends—helps people meet their behaviour change, health, and weight‐loss goals following surgical intervention among people with higher weight [17, 18]. Yet, research has also found that such support is often lacking entirely [6] or ineffective, in that it either fails to meet a person's needs or is discriminatory (e.g., when one's eating choices are questioned, or they are excluded from physical activities [6]).

Together, this evidence suggests that loneliness and its consequences for effective support present a critical barrier to mental and physical health for people with higher weight. To our knowledge, no loneliness interventions have been evaluated in this population. A small number of interventions have, however, sought to improve social support for people with higher weight, with mixed results. These mostly focused on harnessing existing support networks (e.g., partners, family members) to improve the effectiveness of other weight‐loss or disease management interventions (e.g. [17]). Beyond the management of weight, however, G4H is a promising intervention for targeting loneliness, with benefits for mental health. G4H is a brief, evidence‐based group psychotherapeutic intervention designed to focus on building and sustaining positive social connection and group‐based belonging [19, 20]. G4H also supports people to distinguish helpful relationships from those that might be more harmful (e.g., because they are a source of discrimination), and learn strategies to better manage the latter. G4H is informed by the social identity approach to health, which argues that social group memberships (e.g., family and friends, professional groups, community and interest groups, etc.) support good health when we subjectively identify with these groups [21]. These social group memberships can provide important psychological resources for supporting health, including social support, a sense of belonging, and self‐efficacy [22].

In three clinical trials with people experiencing loneliness and clinical depression or anxiety, G4H has been found to be effective in reducing loneliness and depression compared to (a) a matched wait‐list control, (b) treatment‐as‐usual, and (b) dose‐controlled group Cognitive Behaviour Therapy (CBT) [19, 23, 24]. G4H has been shown to be acceptable and feasible to a variety of populations experiencing loneliness and depression [25], and may be particularly beneficial for people with severe loneliness [26]. The program has also been adapted successfully for specific populations at risk of social disconnection (e.g., retirees and people in substance use recovery) [27, 28]. Based on this evidence, G4H shows promise as an effective approach for reducing loneliness, bolstering effective sources of social support, and improving mental health among people with higher weight.

The current study was the first controlled evaluation of g4h among people with higher weight. We investigated program impact from pre‐intervention (T0) to post‐intervention (T1) and 4‐month follow‐up (T2), relative to a matched control sample. Primary outcomes were loneliness and well‐being. Secondary outcomes were social support for behaviours including healthy eating, physical activity, and weight‐management (referred to hereon as weight‐related social support), depression, and eating disorder symptoms (two of the most common mental health concerns reported by people with higher weight) [5]. We hypothesised significant improvements in primary and secondary outcomes from T0 to T1 and T2 among participants who received G4H, relative to the matched control group.

## 2 Methods

### 2.1 Participants

**Figure 1:**
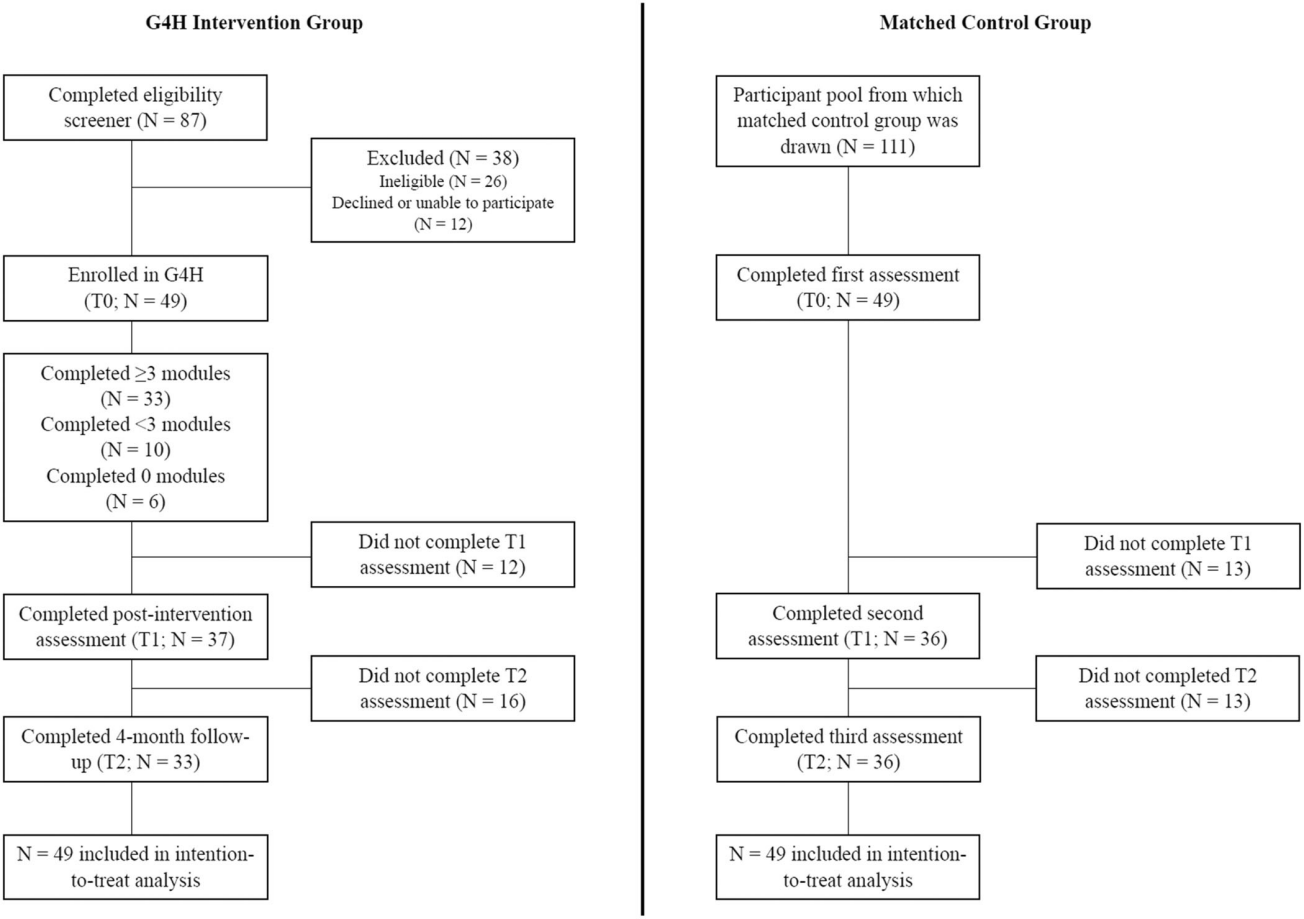
Flow diagram showing participant recruitment and progress in the study.

Figure 1 shows participant recruitment and progress. The two groups of participants were recruited between May 2022 and August 2022. First, adults with a BMI ≥ 30 kg/m^2^ living in Australia and able to attend the authors' institution were invited to complete a screening questionnaire if they were interested in building supportive social networks and connecting with other people with higher weight. A variety of recruitment strategies were used, including paid advertising in local news outlets and on social media, and postcards displayed at local healthcare and obesity management services. After completing the screening questionnaire, individuals who were initially deemed eligible were followed up via phone by a (trainee‐)psychologist to confirm eligibility. Suicide risk assessments were completed for participants who reported experiencing suicidal ideation over the past fortnight (based on Item 9 of the Patient Health Questionnaire 9; PHQ‐9; see Supporting Information [29]), with supervision from an experienced clinical psychologist. Current substance dependence was also assessed at this time. Individuals with high suicide risk (e.g., severe suicidal ideation, with no external supports) and/or substance dependence were deemed ineligible and appropriate referrals were provided. Eligible participants were invited to participate in the g4h program and evaluation.

Inclusion criteria were: (a) being at least 18 years of age, (b) having a BMI ≥ 30 kg/m^2^, and (c) having sufficient English language skills to understand and join in group discussions. BMI was determined using self‐reported height and weight. Exclusion criteria were: (a) high suicide risk, (b) severe psychological symptoms that would interfere with ability to participate in the intervention (e.g. psychotic episode, substance dependence/intoxication), and (c) severe neurological conditions or intellectual impairment that would limit capacity to engage with the intervention. Group facilitators monitored participants throughout the intervention for changes that might affect their eligibility. Eligibility status did not change for any participants.

A target sample size of 50 participants was set based on the pre‐registered power analysis which suggested that with a 60% retention rate (the minimum achieved in previous G4H trials [24]), 28 people at follow‐up would provide 80% power to detect a medium effect size at an alpha level of 0.05. Of the 89 individuals who completed the screening questionnaire for the intervention condition, 49 were deemed eligible, provided written consent, and were allocated to a group. Full participant demographic characteristics and baseline scores for primary and secondary outcomes are provided in Table 1. Among the 49 participants in the intervention group, 38 (77.6%) identified as women; age ranged from 22 to 71 years (*M* = 47.96 years); BMI ranged from 30.69 to 70.70 kg/m^2^ (*M* = 41.68 kg/m^2^). Almost three‐quarters of participants had received a prior mental health diagnosis from a mental health professional (*n* = 36, 73.5%). On average, participants reported moderate clinical levels of depression (PHQ‐9) at T0.

**Table 1:** Participant demographic and baseline characteristics among the intervention group and matched control group.

	G4H intervention group (*n* = 49)	Matched control group (*n* = 49)
	*n* (%)	
Gender		
Man	5 (10.2)	6 (12.2)
Woman	38 (77.6)	41 (83.7)
Nonbinary	1 (2.0)	2 (4.1)
Preferred not to say	5 (10.2)	0 (0.0)
Ethnicity		
Aboriginal and/or Torres Strait Islander	1 (2.0)	0 (0.0)
Asian	2 (4.1)	1 (2.0)
Hispanic/Latina/o	1 (2.0)	0 (0.0)
Pacific Islander	0 (0.0)	1 (2.0)
White	33 (67.3)	40 (81.6)
Self‐specified	4 (8.2)	6 (12.2)
Preferred not to say	8 (16.3)	1 (2.0)
Education		
High school	2 (4.1)	4 (8.2)
Trade certificate or equivalent	8 (16.3)	6 (12.2)
Some university	5 (10.2)	5 (10.2)
Bachelor's degree	17 (34.7)	20 (40.8)
Postgraduate degree	12 (24.5)	13 (26.5)
Other/preferred not to say	5 (10.2)	1 (2.0)
Previous mental health diagnosis		
Yes	36 (73.5)	31 (63.3)
No	6 (12.2)	16 (32.7)
Unsure	5 (10.2)	1 (2.0)
Preferred not to say	2 (4.1)	1 (2.0)
	*M* (SD)	
Age	47.96 (12.08)	43.82 (13.71)
BMI	41.68 (9.14)	40.37 (8.76)
PHQ‐9 Depression T0	13.43 (6.23)	12.27 (6.44)
Loneliness	53.53 (10.77)	50.20 (14.73)
Well‐being	18.91 (3.24)	19.56 (3.76)
Weight‐related social support	3.04 (0.55)	3.21 (0.50)
Depression symptoms	18.04 (11.14)	17.51 (12.47)
Eating disorder symptoms	17.00 (6.39)	14.96 (6.75)

To maximise retention and data quality, participants were offered a voucher (AU$30) following completion of each assessment at post‐intervention (T1) and 4‐month follow‐up (T2). Of the 49 participants in the intervention group, 37 (75.5%) completed the T1 assessment and 33 (67.3%) completed the T2 assessment, a retention rate that exceeded our conservative target and was on par with previous trials of G4H and psychotherapy more generally [30]. In line with previous trials, program completion was defined as attending a minimum of three modules [25]; *n* = 33 participants (67.3%) met this threshold. The median and modal attendance rates were four and five modules, respectively. A post hoc analysis assessed whether the number of modules completed was related to any baseline demographic variables or our primary or secondary measures. No significant relationships were identified.

To yield a matched (non‐randomised) control comparison, a second participant group was identified and drawn from a separate larger longitudinal study with adults with a BMI ≥ 30 kg/m^2^. Participants were recruited through the crowdsourcing platform Prolific and received remuneration of £3 for each assessment timepoint they completed. From this larger study (*N* = 111), a subgroup of 49 participants living in Australia were matched to the intervention group (using the intention‐to‐treat sample, *n* = 49) on baseline characteristics of age, gender, BMI, ethnicity, education level, history of a mental health diagnosis, and T0 depression (PHQ‐9). The PHQ‐9 was used to match participants on baseline depression symptom severity, rather than the DASS‐21 Depression subscale, to retain an outcome measure that was not derived from baseline sample characteristic indicators. There were no significant differences between the intervention and matched control group on these T0 variables. Among the 49 participants in the matched control group, 41 (83.7%) identified as women, with 31 (63.3%) having had a previous mental health diagnosis, and PHQ‐9 scores indicated participants on average were experiencing moderate levels of depression at T0. They were aged between 20 and 85 years (*M* = 43.82 years) and had a BMI range of 31.89 to 68.68 kg/m^2^ (*M* = 40.37 kg/m^2^). Retention rates were similar to those of the intervention group: 36 (73.5%) completed the T1 and T2 assessments.

### 2.2 Study Design

This study was a controlled (non‐randomised) clinical trial with a 4‐month follow‐up. The study procedures were registered on the Australian New Zealand Clinical Trials Registry (Trial ID: ACTRN12622000304730) on 17 February 2022 and were approved by the Australian National University Human Research Ethics Committee (protocol #2022/132). All participants provided written informed consent. A total of seven G4H groups were conducted with a mean of seven participants in each.

### 2.3 Procedure

Outcomes were assessed at three timepoints for all participants: T0, T1, and T2. Both the intervention and matched control groups were invited to complete the T1 assessment 2 months after T0 (following program completion for the intervention group), and the T2 assessment 4 months after T0. All commencing participants in the intervention group were invited to complete the T1 and T2 assessments, including those who had discontinued participation in G4H. At T0, additional measures and demographic questions were included to assist with describing the sample, determining eligibility, and enable matching: age, gender, height, weight, ethnicity, education level, history of mental health diagnosis and PHQ‐9.

#### 2.3.1 The Groups 4 Health Intervention

The G4H program [20] comprises five modules (approximately 75 min each) and is delivered in groups of five to eight people. Participants learn about the importance of social connectedness and group‐based belonging for health (Module 1) and develop a visual ‘social identity map’ to establish current group belonging (Module 2). Participants then develop goals and strategies for strengthening connection with existing groups (Module 3) and joining new groups (Module 4). Modules 1–4 are delivered weekly, with a final module/booster session delivered 1 month later, where participants celebrate their progress and troubleshoot any setbacks (Module 5). Consistent with previous trials, participants who missed a session were offered a catch‐up session and were recorded as having completed the module if they took this opportunity [25]. Before recruitment, G4H was reviewed through a coproduction process with a member of the research team with lived experience of higher weight, to ensure that the content was non‐stigmatising and suitable for people with higher weight. Only minimal revisions to terminology were required.

Groups were facilitated in pairs by trainee psychologists completing a professional psychology graduate program. In addition to their standard training in supportive counselling and risk assessment, all facilitators were provided with 1 day of training in G4H and 1 h/week supervision by a registered clinical psychologist with expertise in G4H. Adherence to the manualised program was maintained via direct observation and weekly supervision.

### 2.4 Measures

#### 2.4.1 Primary Outcomes

As per the trial registration, primary outcomes comprised loneliness and well‐being. *Loneliness* was assessed using the 20‐item Revised UCLA Loneliness Scale [31]. This is perhaps the most widely used and validated measure of loneliness [32]. Participants indicated how often they felt the way described, on a 4‐point scale (1 = never, 4 = often; e.g., ‘People are around me but not with me’). Ten items were reverse scored. The scale had high internal consistency: *ɑ*_T0_ = 0.95, *ɑ*_T1_ = 0.95, *ɑ*_T2_ = 0.96. *Well‐being* was assessed using the 7‐item Short Warwick‐Edinburgh Mental Well‐being Scale (SWEMWBS) [33], which has been extensively validated in clinical and nonclinical samples (e.g. [34]). Well‐being, rather than an indicator of mental ill‐health, was chosen as a primary outcome because the current trial did not limit recruitment to those experiencing elevated symptoms of mental ill‐health. Participants indicated how often they had experienced feelings or thoughts in the past 2 weeks, on a 5‐point scale (1 = none of the time, 5 = all of the time; e.g., ‘I've been feeling relaxed’). The SWEMWBS had good internal consistency: *ɑ*_T0_ = 0.88, *ɑ*_T1_ = 0.89, *ɑ*_T2_ = 0.90.

#### 2.4.2 Secondary Outcomes

Secondary outcomes comprised weight‐related social support, depression symptoms, and eating disorder symptoms. *Weight‐related social support* was assessed using the recently validated 41‐item Weight‐Related Interactions Scale [35]. Participants indicated how often they had experienced criticism, minimisation/undermining, and collaboration for health behaviour from their social networks in the past 3 months (e.g., ‘Offered me food(s) that I am trying to reduce’) on a 5‐point scale (1 = never, 5 = often). Following reverse scoring, responses to all items were averaged, with higher scores indicative of greater received social support for healthy eating, physical activity, and weight management. The scale had high internal consistency: *ɑ*_T0_ = 0.91, *ɑ*_T1_ = 0.92, *ɑ*_T2_ = 0.90.

*Depression symptoms* were measured using the 7‐item DASS‐21 Depression subscale [36], which has been validated for both clinical and nonclinical samples (e.g. [37]). Participants responded to items on a 4‐point scale (0 = Did not apply to me at all, 3 = Applied to me most of the time; e.g., ‘I felt that life was meaningless’). The subscale had high internal consistency: *ɑ*_T0_ = 0.93, *ɑ*_T1_ = 0.95, *ɑ*_T2_ = 0.96.

*Eating disorder symptoms* were assessed using the 12‐item Eating Disorder Examination Questionnaire Short Form (EDE‐QS) global score [38], which is a validated clinical screener for eating disorders (see also [39]). Participants indicated how often they had experienced symptoms in the past 7 days (e.g., ‘…deliberately trying to limit the amount of food you eat to influence your weight or shape (whether or not you have succeeded)’), using a 4‐point scale (0 = 0 days, 3 = 6‐7 days). The scale had acceptable internal consistency: *ɑ*_T0_ = 0.84, *ɑ*_T1_ = 0.81, *ɑ*_T2_ = 0.74.

Additional health and process outcomes beyond the scope of this project were also assessed, with full details available in the trial registration.

### 2.5 Analysis Plan

Hypotheses were tested using mixed effects repeated measures modelling with full information maximum likelihood to manage missing data and retain all commencing participants for intention‐to‐treat analyses [40]. This approach has been recommended as it retains all available data, provides the most accurate estimate of standard error, and correctly handles interdependence between timepoints within individuals (e.g., [41, 42]). Sample matching and analyses were conducted in R (v4.2.3) using the *MatchIt*, *lme4*, and *emmeans* packages. Fixed effects were specified for time (categorical: T0, T1, T2), group (categorical: intervention group, matched control group), and time × group interaction. A random intercept was specified for participant. As the matched control group did not receive any intervention, therapy group could not be included as an additional random intercept. However, when analyses were conducted with the intervention group only [i.e., where time was the only fixed effect], examination of the interclass correlation coefficients indicated that including therapy group as a second random intercept did not significantly improve the fit of the model for any outcomes. Significant effects of time and time × group interaction were followed up with planned contrasts using estimated marginal means, adjusting for multiple comparisons using the multivariate *t*‐distribution. The intervention was delivered between T0 and T1, and so a linear pattern of improvement across the three timepoints was not necessarily expected. Therefore, interactions were assessed for both linear and quadratic patterns (which may indicate, e.g., improvement from T0 to T1 but no change from T1 to T2).

## 3 Results

### 3.1 Primary Outcomes

**Figure 2:**
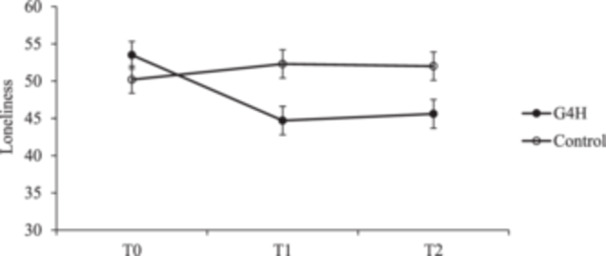
Change in loneliness severity over time in the G4H intervention group and the matched control group. *Note*: Bars indicate standard error based on estimated marginal means. Control, matched control group; G4H, Groups 4 Health group.

Full results are presented in Table 2. Results revealed significant linear (*t*(150) = −6.12, *SE* = 1.59, *p* < 0.001) and quadratic (*t*(148) = 4.35, *SE* = 2.78, *p* < 0.001) time × group interactions on loneliness (see Figure 2). Planned contrasts found that loneliness decreased significantly in the intervention group from T0 to T1 (*t*(151) = −8.04, *SE* = 1.10, *p* < 0.001, *d* = −0.72) and from T0 to T2 (*t*(151) = −6.90, *SE* = 1.15, *p* < 0.001, *d* = −0.66). Loneliness did not significantly change over time in the matched control group (*p*s ≥ 0.106).

**Table 2:** Results from mixed effects repeated measures modelling of the hypotheses.

	*β*	SE	*t* statistic	*df*	*p* value
Loneliness					
Group	0.25	0.20	1.30	113.38	0.196
Time					
T1 vs. T0	0.16	0.08	1.94	145.22	0.054
T2 vs. T0	0.14	0.08	1.67	145.22	0.097
Group × Time					
T1 vs. T0	−0.83	0.12	−7.14	145.75	<0.001
T2 vs. T0	−0.74	0.12	−6.21	145.91	<0.001
Well‐being					
Group	−0.19	0.19	0.99	130.02	0.326
Time					
T1 vs. T0	−0.12	0.11	−1.06	147.74	0.291
T2 vs. T0	−0.18	0.11	−1.63	147.74	0.105
Group × Time					
T1 vs. T0	0.90	0.15	5.87	148.94	<0.001
T2 vs. T0	0.89	0.16	5.65	149.33	<0.001
Weight‐related social support					
Group	−0.36	0.20	−1.78	123.72	0.078
Time					
T1 vs. T0	−0.09	0.11	−0.89	144.47	0.374
T2 vs. T0	−0.08	0.11	−0.71	144.47	0.477
Group × Time					
T1 vs. T0	0.44	0.15	2.96	145.83	0.004
T2 vs. T0	0.69	0.15	4.52	146.19	<0.001
Depression symptoms					
Group	0.06	0.19	0.31	125.94	0.759
Time					
T1 vs. T0	0.17	0.10	1.64	146.72	0.103
T2 vs. T0	0.26	0.10	2.54	146.72	0.012
Group × Time					
T1 vs. T0	−0.73	0.15	−4.95	147.82	<0.001
T2 vs. T0	−0.79	0.15	−5.23	148.16	<0.001
Eating disorder symptoms					
Group	0.34	0.20	1.68	136.18	0.095
Time					
T1 vs. T0	0.07	0.128	0.55	144.48	0.582
T2 vs. T0	−0.09	0.13	−0.73	144.48	0.466
Group × Time					
T1 vs. T0	−0.63	0.18	−3.45	145.57	0.001
T2 vs. T0	−0.64	0.19	−3.40	146.31	0.001

**Figure 3:**
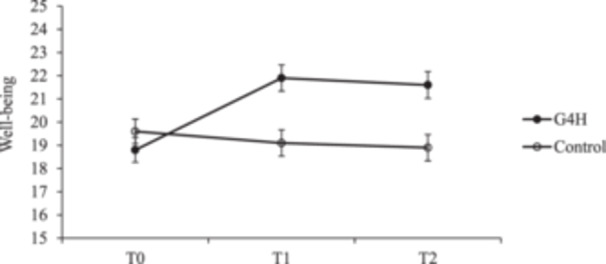
Change in well‐being over time in the G4H intervention group and the matched control group. *Note:* Bars indicate standard error based on estimated marginal means. Control, matched control group; G4H, Groups 4 Health group.

Significant linear (*t*(153) = 5.56, *SE* = 0.62, *p* < 0.001) and quadratic (*t*(149) = −3.28, *SE* = 1.08, *p* = 0.001) time × group interactions were also found for well‐being (see Figure 3). Planned contrasts found that loneliness decreased significantly in the intervention group from T0 to T1 (*t*(154) = 7.12, *SE* = 0.43, *p* < 0.001, *d* = 0.87) and from T0 to T2 (*t*(154) = 6.15, *SE* = 0.45, *p* < 0.001, *d* = 0.80). No significant change over time in well‐being was observed in the matched control group (*p*s ≥ 0.196).

### 3.2 Secondary Outcomes

Analyses revealed a significant linear interaction for weight‐related social support, *t*(151) = 4.45, *SE* = 0.08, *p* < 0.001 (see Figure S1). The intervention group, on average, reported a significant increase in weight‐related social support from T0 to T1 (*t*(152) = 3.22, *SE* = 0.06, *p* = 0.003, *d* = 0.37) and T0 to T2 (*t*(153) = 5.45, *SE* = 0.06, *p* < 0.001, *d* = 0.68). Weight‐related social support did not change significantly over time in the matched control group (*p*s ≥ 0.593).

A significant effect of time (T2 vs. T0) for depression was observed. This effect was qualified by a significant linear (*t*(152) = −5.15, *SE* = 1.91, *p* < 0.001) and quadratic (*t*(149) = 2.50, *SE* = 3.34, *p* = 0.013) time × group interaction (see Figure S2). Depression symptoms decreased significantly in the intervention group from T0 to T1 (*t*(153) = −5.26, *SE* = 1.32, *p* < 0.001, *d* = −0.61) and from T0 to T2 (*t*(153) = −4.73, *SE* = 1.38, *p* < 0.001, *d* = −0.58). The intervention group reported an average reduction in depression of 6.6 points at T2, moving from the moderate (*M* = 18.3) to mild (*M* = 11.7) range of symptom severity, which was clinically significant [43]. In contrast, the matched control group on average reported a significant increase in depression symptoms from T0 to T2 (*t*(151) = 2.50, *SE* = 1.31, *p* = 0.026, *d* = 0.29).

A significant linear interaction was also found for eating disorder symptoms, *t*(154) = −3.35, *SE* = 1.19, *p* = 0.001 (see Figure S3). Planned contrasts revealed that, in the intervention group, eating disorder symptoms significantly decreased from T0 to T1 (*t*(154) = −4.25, *SE* = 0.82, *p* = 0.001, *d* = −0.58) and T0 to T2 (*t*(155) = −5.28, *SE* = 0.87, *p* < 0.001, *d* = −0.77). The clinical cut‐off on the EDE‐QS for a probable eating disorder is 15 (Prnjak et al. [39]). Average scores in the intervention group changed from 17.1 (above the clinical cut‐off) to 12.5 (below the clinical cut‐off). No significant change over time in eating disorder symptoms was observed in the matched control group (*p*s ≥ 0.702).

## 4 Discussion

This controlled evaluation of G4H in people with higher weight provided support for our pre‐registered hypotheses. Specifically, we found that participants in the G4H intervention group reported, on average, clinically significant improvements in loneliness, depression, and eating disorder symptoms relative to baseline that were sustained at the 4‐month follow‐up (2 months post‐intervention). The intervention group also reported significant improvements in their well‐being and the support they received from their social networks for healthy eating, physical activity, and weight management. Weight‐related social support and eating disorder symptoms continued to improve at a steady rate from post‐intervention to the 4‐month follow‐up. Improvements in loneliness, well‐being, and depression primarily occurred between pre‐intervention and post‐intervention, and were maintained from post‐intervention to follow‐up. All observed effect sizes were medium to large, suggesting that the benefits of this brief intervention for people with higher weight in this study were substantial. Effect sizes for loneliness and depression were comparable to previous G4H trials [19, 24], although this was the first evaluation of any loneliness intervention among a higher‐weight population specifically.

By contrast, participants in the matched control group reported, on average, no significant changes over time in loneliness, well‐being, eating disorder symptoms, or weight‐related social support, and a *significant increase* in depression from the pre‐intervention to follow‐up timepoints. Together, these findings provide support for our hypotheses and strong early evidence that G4H is a promising intervention for addressing psychosocial barriers to good health experienced by people with higher weight—namely loneliness, ineffective social support, and mental ill‐health [2, 5, 6].

This research has implications for both research and practice. First, the findings suggest that the unmet social and mental health needs of people with higher weight are substantial. The current trial did not limit recruitment to those who were experiencing loneliness or mental ill‐health. Nevertheless, baseline assessments indicated that participants in both groups were, on average, experiencing high levels of loneliness (e.g., more than one standard deviation above the mean of control groups in previous RCTs targeting loneliness [44, 45]. On average, participants were also experiencing clinically significant levels of depression and eating disorder symptoms at baseline [39, 41], and the majority had a history of at least one formal mental health diagnosis. These baseline characteristics exemplify the significant social connection and mental health needs of people with higher weight.

The present findings indicate that G4H may be an appropriate program to tackle these social and mental health needs for higher‐weight people. Our findings support the efficacy of delivering G4H as a solo intervention, however, future evaluations of G4H might investigate the added benefits that G4H may have when delivered alongside other health‐related (e.g., behaviour change) interventions. Additionally, future research should explore the mechanisms through which G4H supports improvements in loneliness and mental health among people with higher weight. Candidate mechanisms include increased access to psychological resources (namely effective social support), gain in new supportive groups, improved capability to manage challenging groups, social identification with the therapy group, and connecting with people with lived experience of higher weight and shared experience of discrimination [24, 25, 46]. Understanding the active ingredient will enable future applications of G4H to maximise the positive impact of the program on loneliness and health outcomes among people with higher weight.

### 4.1 Strengths and Limitations

A strength of the study was that it reached a population with complex health needs and did not exclude people with comorbidities. Retention rates in the current trial were comparable to previous G4H trials [19, 24], and to psychotherapy more broadly [30]. Another strength was the inclusion of a matched control group, providing further confidence that the observed improvements in primary and secondary outcomes were attributable to the G4H intervention and not naturally occurring changes over time. However, it was not feasible for participants to be randomly assigned to condition, with participants in the matched control group originally recruited for a separate larger study. Therefore, it is possible that these two samples may have differed on unmeasured characteristics that we were unable to control. As the matched control group did not participate in any group therapy, it is also possible that the observed effects were attributable to group therapy participation more generally, rather than G4H specifically. The previous phase III RCT of G4H provides some confidence that this is not the case, given G4H was found in a different population to be as effective as dose‐controlled group CBT in reducing depression symptoms, and more effective in reducing loneliness. Nevertheless, the next phase of evaluation should use an RCT design to provide a more rigorous test of the benefits of G4H for people with higher weight.

## 5 Conclusion

People with higher weight face many barriers to good health that are not directly weight related. In particular, loneliness and challenging social networks can cause significant distress and mental and physical ill‐health, but are rarely a focus of health interventions. The findings from the current study suggest that empowering people with higher weight with the knowledge and skills to build meaningful supportive group connections has a positive influence on their health. In this, G4H offers a novel approach to counter some of these psychosocial barriers to health that people with higher weight so often face.

## Author Contributions

**Joanne A. Rathbone:** conceptualisation; methodology; formal analysis; writing–original draft; writing–review and editing; funding acquisition; project administration. **Tegan Cruwys:** conceptualisation; methodology; writing–review and editing; supervision; validation. **Kate A. B. Western:** methodology; data curation; project administration; writing–review and editing. **Jessica L. Donaldson:** writing–review and editing; project administration; data curation. **Catherine Haslam:** writing–review and editing; validation. **Elizabeth Rieger:** writing–review and editing; conceptualisation; validation; methodology. **Fiona Tito Wheatland:** methodology; data curation; validation; writing–review and editing. **Paul Dugdale:** conceptualisation; writing–review and editing; validation.

## Ethics Statement

The study was approved by the Australian National University Human Research Ethics Committee (#2022/132).

## Conflicts of Interest

Two of the eight authors (TC and CH) are developers of the Groups 4 Health program. These authors have an academic, rather than commercial, interest in the program, with Oxford University Press holding the associated Intellectual Property. The other authors have no interests to declare.

## Supporting information

Supporting information.

## Data Availability

The data sets generated and analysed during the current study are available in the Open Science Framework repository, https://osf.io/9b3cs/.
