# Supplementary material for: Tackling Loneliness, Ineffective Social Support, and Mental Ill‐Health Among People With Higher Weight
Source: Health Expect. 2025 Mar 27;28(2):e70192. doi: 10.1111/hex.70192 (PMC11950156; doi:10.1111/hex.70192)
Supplement: Supplementary file 1 — Supporting information. [file HEX-28-e70192-s001.docx]

**A controlled trial of an intervention to tackle** **loneliness, ineffective social support, and mental ill-health among people with higher weight**

**Supplementary Materials**

**Patient Health Questionnaire (PHQ-9)**

The Patient Health Questionnaire (PHQ-9) (1) was used to screen for suicide ideation and to provide a baseline indicator of depression symptom severity at T0. Participants responded to 9 items on a 4-point Likert scale (0=not at all, 3=nearly every day), based on symptoms they had experienced over the last two weeks (e.g., “Little interest or pleasure in doing things”). Item 9 was used to screen participants for suicidality: “Thought you would be better off dead or of hurting yourself in some way”. The scale had good internal consistency, ɑ=.88.

**Figure S1**

Change in weight-related social support over time in the G4H intervention group and the matched control group.

*Note.* Bars indicate standard error based on estimated marginal means. G4H: Groups 4 Health group; Control: matched control group.

**Figure S2**

Change in depression symptoms over time in the G4H intervention group and the matched control group.

*Note.* Bars indicate standard error based on estimated marginal means. G4H: Groups 4 Health group; Control: matched control group.

**Figure S3**

Change in eating disorder symptoms over time in the G4H intervention group and the matched control group.

*Note.* Bars indicate standard error based on estimated marginal means. G4H: Groups 4 Health group; Control: matched control group.

**References**

1. Kroenke K, Spitzer RL, Williams JB. The PHQ-9: Validity of a brief depression severity measure. J Gen Intern Med [Internet]. 2001 Sep;16(9):606–13. Available from: http://dx.doi.org/10.1046/j.1525-1497.2001.016009606.x
